# A narrative review of refugee & asylum seekers’ transitions into & experiences of working in the United Kingdom National Health Service

**DOI:** 10.1186/s12913-023-09606-1

**Published:** 2023-06-13

**Authors:** Derek David Truong Farnham, Ross Goldstone

**Affiliations:** 1 National Health Service Greater Glasgow and Clyde West Glasgow Ambulatory Care Hospital, Dalnair St, Glasgow, G3 8SJ UK; 2 NHS England, 3 Piccadilly Place, Manchester, M1 3BN UK

**Keywords:** Refugee and asylum seeker healthcare professionals, Refugee and asylum seeker doctors, Barriers, Challenges, Employment, Language

## Abstract

**Background:**

The United Kingdom (UK) has a significant and rising population of refugees and asylum seekers, including many who have previously worked as healthcare professionals. Evidence shows they have struggled to join and successfully work in the UK National Health Service (NHS) despite initiatives designed to improve their inclusion. This paper presents a narrative review based on research surrounding this population to describe the barriers that have impeded their integration and possible ways to overcome them.

**Methods:**

We conducted a literature review to obtain peer-reviewed primary research from key databases (PubMed, Web of Science, Medline, EMBASE). The collected sources were individually reviewed against predetermined questions to construct a cohesive narrative.

**Results:**

46 studies were retrieved, of which 13 satisfied the inclusion criteria. The vast majority of literature focussed on doctors with minimal research on other healthcare workers. Study review identified numerous barriers impeding the integration of refugee and asylum seeker healthcare professionals (RASHPs) into the workforce that are unique from other international medical graduates seeking employment in the UK. These include experiences of trauma, additional legal hurdles and restrictions on their right to work, significant gaps in work experience, and financial difficulties. Several work experience and/or training programs have been created to help RASHPs obtain substantive employment, the most successful of which have involved a multifaceted approach and an income for participants.

**Conclusions:**

Continual work towards improving the integration of RASHPs into the UK NHS is mutually beneficial. Existing research is significantly limited in quantity, but it provides a direction for future programs and support systems.

**Supplementary Information:**

The online version contains supplementary material available at 10.1186/s12913-023-09606-1.

## Background

This research article examines existing literature on the experiences of and challenges faced by refugee and asylum seeker healthcare professionals (RASHPs) in the United Kingdom (UK) when entering or working in the National Health Service (NHS). Recent decades have seen the global population of forcibly displaced people increase, with the United Nations High Commission on Refugees reporting that, in 2021, there was a total of 84 million [1]. Whilst most are internally displaced within their country of origin, refugees are estimated to constitute 26 million (31%) of all forcibly displaced peoples, with a further 4.4 million seeking asylum in countries across the world [1]. Displaced persons predominantly remain in the region of displacement. Yet, in the past decade developed countries have seen an increase in the number of asylum applications and public interest in such migratory patterns, despite overall refugee populations in developed countries remaining low relative to other Low- and Middle-Income Countries (LMICs). The UK is one such nation – at the end of 2020, the United Nations High Commission on Refugees (2020) reported its current refugee population at 132,349, with a further 77,245 pending asylum applications and 4,662 stateless persons currently residing in the country [2].

Those displaced because of persecution, war, or violence, and forced to flee to the UK typically possess a range of knowledge and skills, which they may be unable to utilise for a number of structural barriers. This is especially the case in fields including medicine, nursing, and other allied health professions where national regulations for entry present an additional barrier to entry. For example, depending on occupation, some must meet the accreditation requirements of professional associations, such as the General Medical Council (GMC) or Nursing and Midwifery Council (NMC), which include a combination of professional competence and, where appropriate, English language testing. These tests of professional and linguistic competence aim to ensure that internationally educated health professionals, such as RASHPs, meet the standards expected of all practitioners in the NHS. However, as the findings of this narrative review will indicate, highly qualified RASHPs encounter a range of challenges and issues when attempting to return to professional practice in the UK. Some of these issues are shared with the wider internationally educated health professional community, whilst others present unique barriers to entry for refugee health professionals.

Therefore, this research is well positioned to inform current and future concerns of policymakers working in health and social care in the UK, as well as those with responsibility for the healthcare workforce in other developed countries with not insignificant numbers of refugee health professionals. It aims to answer the following research questions:

1. What common challenges and barriers do RASHPs encounter during their transition into the NHS?
2. What common challenges and barriers do RASHPs encounter when working within the NHS?
3. How can RASHPs be better supported during their transition into and whilst working within the NHS?

The remainder of this article progresses as follows. Firstly, the methodological approach adopted in this evidence review is outlined. This is followed by the study’s results, organised as key themes identified in the literature reviewed. Thereafter, the study’s results are discussed with reference to (a) the gaps in the existing evidence and the (b) significance of the findings for policymakers and practitioners. In synthesising the existing research to date on the experiences and challenges faced by UK-based health professionals when entering the NHS, this article will conclude by suggesting areas for future research and evidence-informed recommendations for how to effectively support refugee health professionals to return to practice.

## Methods

To determine the barriers to RASHP integration into the NHS along with possible policy solutions to address these, we conducted a narrative review of currently available literature identified by a systematic search of key databases organised by the research questions of this paper.

### Search strategy

The electronic databases, Ovid MEDLINE®, Ovid EMBASE, Web of Science Core Collection, and PubMed, were searched for study retrieval. Initial broad searches were used to establish the range of literature that existed involving RASHPs and to guide the creation of specific search protocols. Based on these searches, we chose the following terms to identify relevant studies in each database: “refugee doctor*”, “refugee nurse*”, “refugee pharmacist*”, “refugee dentist*”, “refugee biomedical scientist*”, “refugee physiotherapist*”, or “refugee healthcare professional*” in combination with “United Kingdom”, or “National Healthcare System” in their title, abstract, or as a key word. Papers not in the English language were also filtered out in the searches. Given the limited pre-existing research in this topic, year of publication was not included in the protocols. Individual search protocols can be found under Appendix A.

### Study selection

The resulting articles from each database were cross-referenced to have duplicates removed, then screened against the following predetermined set of criteria. Articles were included if they focussed on RASHPs in the context of joining and working within the UK/NHS. Articles were excluded if they lacked primary data, such as editorials and news articles, or if they were not published in the English language. The list of articles retrieved was screened against these inclusion/exclusion criteria first based on title and abstract, then as full-text articles. Articles for which full access could not be gained were also excluded.

### Narrative synthesis

Articles were first grouped by type of study, then each selected paper was individually reviewed by the lead author to extract all relevant data to answer the following questions:

1. What barriers to RASHPs face regarding their integration within the NHS:
  - at a personal level?
  - at an interpersonal level?
  - at a structural level?
  - that differ from the barriers that non-RASHP internationally educated health professionals face?
2. What initiatives have been attempted to improve the situation for RASHPs and how successful have they been?
3. What can be done to improve the situation for RASHPs in the future?

The data were then grouped according to research type and their respective conclusions to synthesise a narrative addressing the aims of this paper.

Although some studies had quantitative data components, the lack of consistent variables and data types made a secondary analysis unfeasible. However, relevant quantitative data from such studies were used in the construction of the narrative.

### Patient and public involvement

Patients and the public were not involved in any way whilst conducting this literature review.

## Results

### Results of study selection

**Fig. 1:**
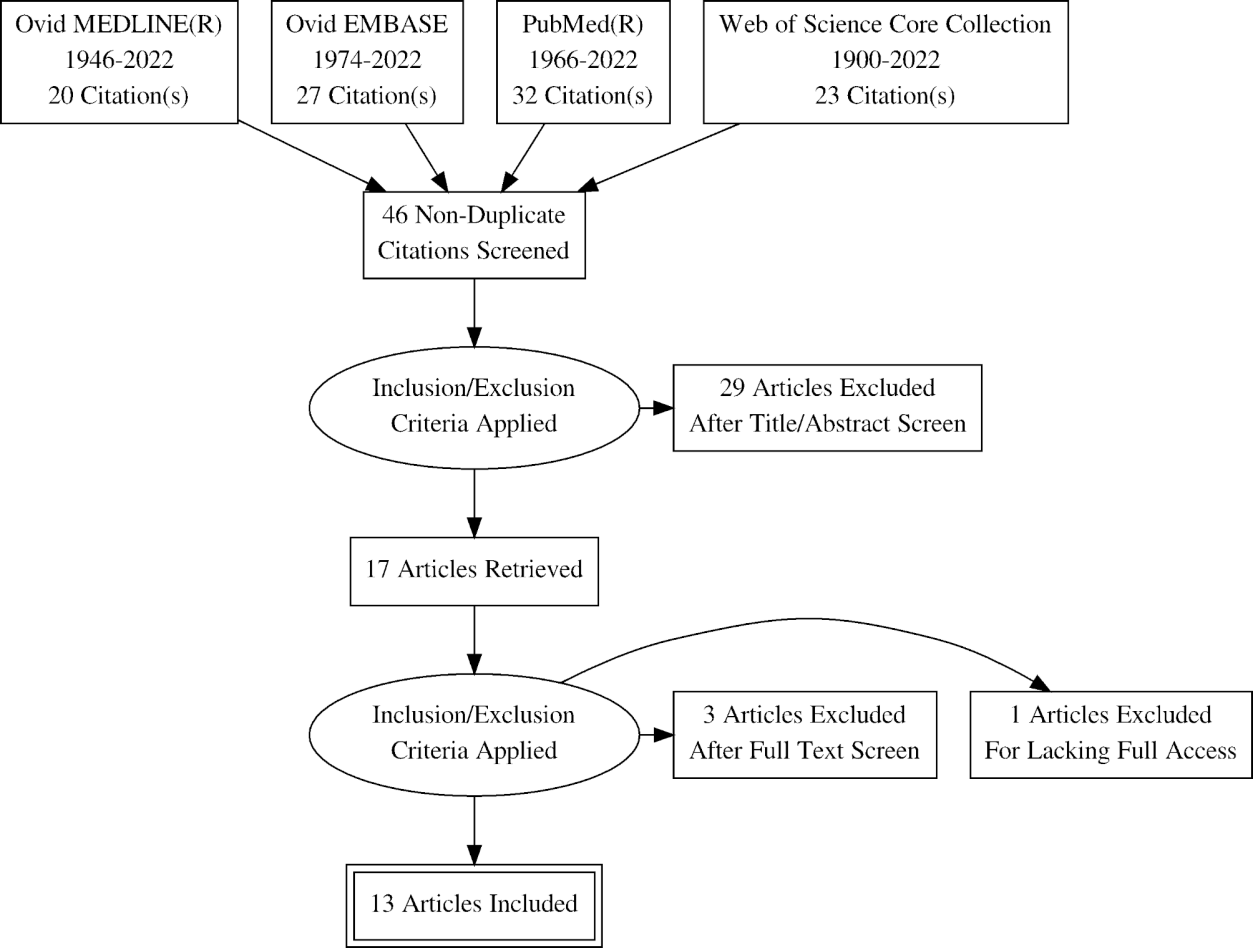
PRISMA flow diagram for literature search

A total of 46 non-duplicate papers were identified by following the designed search protocols for each database. Initial screening of titles and abstracts resulted in 29 papers being excluded, mainly for not being peer-reviewed original research with primary data. Of the 17 remaining papers, four were excluded after full screening, one for lacking full access and the remainder for lacking primary data. Overall, 12 articles were selected for full review. The full process and results of study selection is represented in Table 1; Fig. 1. The full search results can be found in Appendix B.

**Table 1:** Results of Study Selection

Author	Title	Publication Information (Journal, Year)	Area of Focus
Anderson	Medical students and refugee doctors: learning together.	Medical Education, 2007	Initiative
Butt	Integrating Refugee Healthcare Professionals In The UK National Health Service: Experience From A Multi-Agency Collaboration.	Advances in Medical Education and Practice, 2019	Barriers: personal, interpersonal, structural, unique to RASHPs Initiative Improvement
Cohn	Experiences and expectations of refugee doctors.	British Journal of Psychiatry, 2006	Barriers: personal, structural, unique to RASHPs
Eastwood	Re-training refugee and other overseas doctors: re-qualification through the United Examining Board examination.	Clinical Medicine, 2006	Barriers: unique to RASHPs Initiative Improvement
Gavin	Solving the recruitment crisis in UK general practice: Time to consider physician assistants?	Social Policy and Administration, 2002	Improvement
Leblanc	Comparing approaches to integrating refugee and asylum-seeking healthcare professionals in Canada and the UK.	Healthcare Policy, 2013	Barriers: personal, structural
Ong	Investing in learning and training refugee doctors.	Hospital Medicine, 2003	Initiative Improvement
Ong	Helping refugee doctors get their first jobs: the pan-London clinical attachment scheme.	The Clinical Teacher, 2010	Barriers: interpersonal, structural Initiative Improvement
Pietka-Nykaza	‘I Want to Do Anything which Is Decent and Relates to My Profession’: Refugee Doctors’ and Teachers’ Strategies of Re-Entering Their Professions in the UK	Journal of Refugee Studies, 2015	Barriers: personal, structural, unique to RASHPs
Roberts	Tall trees; weak roots? A model of barriers to English language proficiency confronting displaced medical healthcare professionals	Language Teaching Research, 2020	Barriers: structural, unique to RASHPs Improvement
Shah	An evaluation of the CAPS refugee doctor scheme in London - a survey of outcomes.	Education for Primary Care, 2021	Initiative Improvement
Sinclair	Refugee doctors as doctors’ assistants in psychiatry.	Psychiatric Bulletin, 2006	Initiative
Stewart	Refugee doctors in the United Kingdom.	British Medical Journal, 2002	Barriers: personal, interpersonal, structural, unique to RASHPs

### Characteristics of included studies

Seven of the 13 articles included for review were exclusively qualitative [3–9]. Of these, six were interview based [3, 5–9], and one was a narrative study [4]. Two articles were exclusively quantitative [10, 11]. These were both cohort studies focussing exclusively on employment outcomes. The remaining four articles had both qualitative and quantitative components [12–15]. Of these, three were cohort studies that focussed on employment outcomes and survey feedback [12, 13, 15], and one was a mixed methods study involving language assessments and focus groups [14]. All studies focussed at least in part on refugee and asylum seeker doctors, whereas only three included data on other refugee professionals [5, 6, 10]. One focussed on medical students as well as doctors [9], one focussed on teachers as well as doctors [6], one focussed on nurses as well as doctors [5], and one focussed on several kinds of healthcare professionals including doctors [10].

Three of the interview studies focussed on the perspective of RASHPs and their perceived barriers to entering and working in the NHS [3, 6, 8]. One of the interview studies focussed on feedback from colleagues and patients following a RASHP assistantship program in psychiatry [7]. Another focussed on feedback from medical students and refugee doctors who partook in an informal program for both parties to practice for objective structured clinical examinations (OSCEs) [9]. The remaining interview study focussed on the perspective of healthcare industry stakeholders with a role in RASHP integration [5].

The narrative study proposed a novel pathway for employment of RASHPs based on data surrounding their expertise and preferences as well as employment gaps in the NHS [4]. The five cohort studies all aimed to determine the impact of various training and assistantship programs on employment outcomes for RASHP participants [10–13, 15]. The mixed methods study focussed mainly on barriers surrounding English language acquisition and certifications for RASHPs as well as possible solutions [14].

One study focussed on Canada as well as the UK [5], with all others focussing exclusively on the UK.

### Key issues identified in the literature

This section discusses the results of the narrative review conducted. It is organised according to the questions outlined in the methods section in relation to this paper’s objectives, specifically: [1] barriers to successful employment, [2] attempts to overcome barriers, [3] remaining areas for improvement.

### Barriers to integration within the NHS

Multiple papers made the distinction between barriers to employment due to legal structures, versus those related to personal circumstances [6, 10]. Restrictions on their right to work within the UK, which can last from months to years, create uncertainties regarding status and right to remain in the country [6].^1^ RASHPs have also described the difficulties encountered when converting qualifications obtained elsewhere to UK standards [6]. RASHPs sometimes struggle to prove their qualifications at all, having lost them amid their often sudden and unexpected uprooting [6]. Experiencing persecution and trauma in their homeland, as well as facing negative representations in media and public discourse within the UK were described as significant personal barriers for RASHPs [5, 10]. One study found that of the 29 RASHP participants, 26 scored symptoms on the Post Traumatic Stress Disorder (PTSD) scale, of which three scored above the cut-off for the clinical threshold of PTSD [12].

In two qualitative interview studies focused on medical professionals, the language and medical qualification examinations mandated by the GMC^2^ were cited as some of the largest and most problematic barriers to gaining work [8, 11]. These were described as a more significant challenge for RASHPs in comparison to internationally educated health professionals [8, 11]. First, RASHPs often face a significant gap in practice – on average 4.5 years – and the deskilling and loss of confidence in one’s abilities that comes with it [11]. This is uncommon for non-RASHP internationally educated health professionals, who usually have the opportunity and resources to adequately prepare for the required examinations prior to entering the UK [11]. Some interviewees took issue with the seeming lack of tailoring of the IELTS examination to the specific requirements of medical practice, as well as the fact that they had to pass it despite having been trained in English [8]. In another interview study, the need to balance the stringent requalification requirements with familial and financial responsibilities was cited as an additional barrier to RASHPs when seeking to return to practice in the NHS [3].

Interviewees across several studies stressed the negative psychological impacts of suddenly being undervalued – worsened by their forced gap in practice and deskilling – as well as the discrimination they faced with the label of being a “refugee doctor” [3, 8, 13]. Some RASHPs were recorded as saying they felt they were considered as second class behind non-RASHP internationally educated health professionals [3, 8]. In one study, RASHPs mentioned the lack of an established pathway into medical practice for the unique circumstances of RASHPs as an additional structural barrier [3].

Leblanc et al.’s findings corroborated the barriers identified by RASHPs themselves. Stakeholders noted the financial constraints RASHPs face, with many unable to afford programs for retraining that are currently available in the UK [5]. It was remarked that some RASHPs are forced to abandon their field altogether and take up alternative jobs to support their families, as a result of the barriers they face [5]. The study identified shortcomings with language proficiency testing that aligned with the findings from studies in which RASHPs were interviewed. It mentions that, because of the generic nature of the exams, passing them does not guarantee a level of language proficiency that satisfies employment requirements [5]. Thus, the examinations present a significant barrier to employment for RASHPs, which once surmounted does not necessarily guarantee linguistic competence, adding to difficulties of low confidence previously mentioned.

One study offered data regarding barriers against successful integration experienced within the workplace environment. RASHPs identified cultural adaptations and differences in communication styles, differences in workplace culture, and experiences of racism, sexism, and ageism as difficulties they experienced whilst completing their attachments [13].

Faced with these barriers, Piętka-Nykaza categorised the decision-making process for RASHPs into four strategies:

- Acceptance: Conform to institutional requirements to continue chosen careers in the UK.
- Compromise: Lower aspirations towards other jobs in healthcare or postpone professional career development, often due to financial constraints.
- Ambivalence: No decision, often due to uncertainty regarding their legal status to remain in the UK.
- Withdrawal: Seek employment in other fields entirely, after exhausting all other strategies [6].

### Attempts to overcome barriers

The cohort studies and the narrative study cover several programs over the past two decades that have been trialled to help RASHPs gain substantive employment in the NHS [4, 10–13, 15]. In this case, substantive employment may be defined as a long-term job in a RASHP’s original career, ideally with opportunities for career progression.

Early programs mentioned by Ong and Gavin were designed in the form of unpaid internships, providing work experience in the NHS at a relatively low cost for employers [4, 12]. However, whilst the impact of RASHPs as a workforce was nearly exclusively positive [4, 7, 12], data suggested these programs resulted in subsequent substantive employment in only a minority of cases [12]. Eastwood suggested that more intensive programs could produce better results while still being cost-effective [11]. Of particular significance is the lack of renumeration for RASHPs undertaking internship opportunities of this kind, which may present financial barriers to participation, especially for those with familial commitments.

Studies covering more recent programs that offer a higher degree of support and an income for RASHP participants have been shown to greatly enhance the likelihood of substantive employment directly following their completion [13, 15]. For example, an earlier internship-based program with more limited support provided to RASHPs resulted in about 50% employment within 8 months following completion [12], whereas the most recent Clinical Apprenticeship Scheme (CAPS) resulted in 93% of participants being retained within the NHS [15]. These newer programs were designed specifically for RASHPs, with tailored educational support, linguistic resources, and pastoral care included [13, 15]. Communications skills support was the most frequently cited benefit of the latter programs according to participants who completed them [15]. Their design has been suggested to help address several factors that contribute to differential attainment between minority ethnic and white medical practitioners, such as a lack of social and cultural capital or a reduced sense of belonging among those from minority ethnic background, which may in part underpin their success [15].

Anderson et al. designed an informal OSCE preparation program between medical students and refugee doctors. The refugee doctors who partook in it described benefits both psychologically and related to language skills [9].

### Remaining areas for improvement

Further supporting RASHPs has been noted as a potential “win-win” given the shortage of healthcare workers [5]. General practice – an underemployed area of the NHS [16] – is the most commonly cited specialty of choice amongst RASHPs [15]. Studies suggest that the main avenues towards improving the current situation for RASHPs at a structural level are investing further in the most successful types of programs described above, along with redesigning language assessment tools and better tailoring language training to the individual and their needs for successful employment in the NHS [5, 14].

## Discussion

The results presented above demonstrate that RASHPs experience significant difficulties when attempting to return to clinical practice in the UK NHS. Some of these difficulties are shared with internationally educated health professionals, yet many are unique to RASHPs given the unique conditions they occupy and the discrimination and prejudice they are confronted with [3, 5, 6, 8, 10, 11, 13]. Internationally educated health professionals face numerous challenges when attempting to enter the NHS, from learning new medicolegal frameworks, training systems, and skills guidelines, to navigating cultural differences with work colleagues and patients [10]. These issues are also pertinent for RASHPs, who face them on top of legal restrictions on their mobility and right to work in the UK and the difficulties of having been suddenly and unexpectedly uprooted from their homes [5]. Hence, RASHPs make up a unique and highly varied group of individuals that struggle when attempting to resume their careers as healthcare professionals in the UK.

English language proficiency testing was identified in the results of this paper as a general difficulty faced by RASHPs. This comes as no surprise given that as of 2008, of the more than 1,000 registered refugee doctors on a now unavailable GMC register, only 14% were practicing medicine, 20% had passed all examinations but were not working, and nearly half had yet to pass the IELTS English language proficiency examination [14]. Despite more recent data being unavailable, this does seem to suggest that the first hurdle to returning to practice in the NHS encountered by healthcare professionals – demonstrating English language proficiency – is particularly troublesome. Results thus point to the importance of providing high-quality English language preparation and support to RASHPs who wish to return to clinical practice and contribute to the UK NHS. In light of negative testimony about the general nature of the IELTS examination, it would seem most appropriate to support RASHPs to undertake OET examination preparation, which is a specially designed English language examination designed for healthcare professionals. This point is strengthened by the higher pass rates achieved by those undertaking OET examinations relative to the IELTS. This is an important consideration to be made given the forthcoming austere post-pandemic environment and the need to ensure investments in the healthcare workforce offer value-for-money at the same time as high-quality outcomes.

However, even in cases where English language proficiency was demonstrated and employment is subsequently secured, RASHPs are known to be unlikely to obtain training positions that healthcare workers usually need to progress in their careers [8, 15].

This paper is consistent with Piętka-Nykaza’s classification of the decision-making process for RASHPs and reinforces the idea that the eventual career paths of RASHPs are not simple results of their choices made in a vacuum. Rather, they are responses to personal dilemmas exacerbated by structural conditions encountered at each stage of the retraining and revalidation process, some of which are unique to each RASHP whilst many characterise the general RASHP experience [6]. This finding would suggest the need for a more nuanced support package for RASHPs in the UK wishing to return to clinical practice in the NHS. Yet, unfortunately such an approach towards supporting RASHPs has often not been implemented at an institutional level, as many have cited dissatisfaction with and barriers related to being homogenised as a single group [3, 5, 8, 13]. Indeed, as suggested by Piętka-Nykaza and RASHPs themselves [3, 5, 6, 8, 13], it would appear the most successful interventions in helping RASHPs obtain substantive employment are tailored to the individual, with a consideration of financial needs through paid working opportunities and the inclusion of additional training and pastoral support [13, 15]. That said, that the lack in quantity of research, heterogenous program design, and limited description of program design make the reproduction and comparison of various training programmes more challenging and less reliable.

Additionally, the previously mentioned limited research on the experiences and challenges of RASHPs when transitioning into the NHS does need to be addressed. Only one study focussed on experiences and barriers RASHPs face in the work environment itself. Additionally, despite the focus of this review on refugee and asylum seeker *healthcare professionals* (RASHPs) in general, most papers reviewed focus exclusively on those who are doctors. As such, whilst the term RASHP has been used throughout this review, the lack of research on RASHPs in other healthcare professional groups (e.g., nurses, AHPs) underscores the need for further research involving these groups, particularly given the chronic workforce shortage in these areas (e.g., Buchan, 2019) [17]. With the current refugee and asylum seeker population in the UK likely to increase in the coming years, and in future decades not least in light of the expected disruption caused by climate change and the consequential significant number of ‘climate refugees’ [18], it is highly pertinent and time-sensitive to address the lack of research in this area. Beyond humanitarian arguments for helping refugee populations effectively integrate into destination countries, ensuring that health professionals can retrain to return to practice is of benefit to health systems of both destination and origin countries. In the immediate, the destination country is able to enhance both the volume and diversity of its healthcare workforce with highly educated healthcare professionals. In the long term, many of these healthcare professionals are likely to want to return to their country of origin to help in rebuilding societies ravaged by disaster and war, which the continuity of skill development and the additional global learning experiences gained from working in a universal health coverage health system, such as the NHS, would facilitate.

In a national context of severe workforce challenges facing the NHS [19, 20] and recognition of the importance of global health system strengthening to deliver NHS objectives, the findings of this research offer a timely contribution to existing research on the challenges faced by and how to support RASHPs in the UK. What existing research shows is that RASHPs must to overcome significant barriers to return to practice in the NHS, which add to the general discrimination and prejudice known to be experienced by refugee and asylum seekers in wider society [21]. Future research on the experiences and challenges of RASHPs is an important step to understand how best to support this vulnerable, yet highly skilled, group to effectively integrate into and contribute to UK society. In doing so, more effective programmes can then be designed that offer mutual benefit to all stakeholders involved.

## Conclusions

This research article has examined existing literature on the experiences of and challenges faced by RASHPs in the UK when entering or working in the NHS. Whilst there is a clear need for further research in this area, the findings indicate that significant structural barriers and personal difficulties are faced by RASHPs when returning to practice in the NHS. In response to the workforce pressures currently impacting on the NHS in all areas of clinical practice, the UK Government has announced an intention to support refugee healthcare professionals currently in the country to return to practice. Although this signifies a positive step for both the individual RASHP and the NHS, significantly more financial and pastoral support will be necessary to deliver quality outcomes and value-for-money. However, in making such investments in RASHPs currently in the UK, a ‘win-win’ is possible, where: the RASHP is enabled to return to practice, better integrate into their new society, and support themselves and their dependents; the NHS benefits from acquiring highly skilled health professionals, some of which possess unique skills and experience currently in short supply in the NHS; and for the RASHPs country of origin, where the RASHP chooses to return to their homeland and contribute to health system strengthening, integrating global learning into local services.

## Electronic supplementary material

Below is the link to the electronic supplementary material.

Supplementary Material 1: Appendix A-D

## Data Availability

All data generated or analysed during this study are included in this published article and its supplementary information files.

## Footnotes

1 Refugees entering the UK may apply for “indefinite leave to remain (ILR), providing them eligibility to work, study, and use public services. It usually takes at least six months to receive a decision following an application. Asylum seekers in the UK may only apply for permission to work (in jobs listed on the government’s “shortage occupation list”) after 12 months of awaiting a decision on their claim.

2 These are the International English language Testing System (IELTS), a generalised English language proficiency test; the Occupational English Test (OET), an English language test tailored to the healthcare sector; and the Professional and Linguistic Assessments Board (PLAB), a two-part exam that allows individuals to certify their qualifications to work as a Senior House Officer in a UK NHS hospital setting.

## Abbreviations

UK: United Kingdom
NHS: National Health Service
RASHP: Refugee and asylum seeker healthcare professional
LMIC: Low- and Middle-Income Country
GMC: General Medical Council
NMC: Nursing and Midwifery Council
OSCE: Objective structural clinical examination
PTSD: Post-traumatic stress disorder
CAPS: Clinical apprenticeship scheme
