# Supplementary material for: A narrative review of refugee & asylum seekers’ transitions into & experiences of working in the United Kingdom National Health Service
Source: BMC Health Serv Res. 2023 Jun 13;23:622. doi: 10.1186/s12913-023-09606-1 (PMC10262440; doi:10.1186/s12913-023-09606-1)
Supplement: Supplementary file 1 — Supplementary Material 1: Appendix A-D [file 12913_2023_9606_MOESM1_ESM.docx]

# Appendix A: Search Protocols

**MEDLINER**

Database: Ovid MEDLINE(R) ALL <1946 to March 25, 2022>

Search Strategy:

--------------------------------------------------------------------------------

1 national health service/ (33138)

2 United Kingdom/ (241456)

3 "refugee doctor*".mp. (39)

4 "refugee nurse*".mp. (12)

5 "refugee pharmacist*".mp. (1)

6 "refugee dentist*".mp. (2)

7 "refugee biomedical scientist*".mp. (1)

8 "refugee physiotherapist*".mp. (1)

9 "refugee healthcare professional*".mp. (3)

10 1 or 2 (271761)

11 3 or 4 or 5 or 6 or 7 or 8 or 9 (52)

12 10 and 11 (20)

**EMBASE**

Database: Embase <1974 to 2022 March 25>

Search Strategy:

--------------------------------------------------------------------------------

1 national health service/ (69098)

2 United Kingdom/ (398530)

3 "refugee doctor*".mp. (42)

4 "refugee nurse*".mp. (10)

5 "refugee pharmacist*".mp. (2)

6 "refugee dentist*".mp. (1)

7 "refugee biomedical scientist*".mp. (0)

8 "refugee physiotherapist*".mp. (0)

9 "refugee healthcare professional*".mp. (2)

10 1 or 2 (422017)

11 3 or 4 or 5 or 6 or 7 or 8 or 9 (56)

12 10 and 11 (27)

**PUBMED**

(("refugee doctor*") OR ("refugee nurse*") OR ("refugee pharmacist*") OR ("refugee dentist*") OR ("refugee physiotherapist*") OR ("refugee biomedical scientist*") OR ("refugee healthcare professional*")) AND (("NHS") OR ("United Kingdom") OR ("National Health Service") OR ("UK"))

**Quoted phrases not found:** refugee pharmacist*, refugee dentist*, refugee biomedical scientist*, refugee physiotherapist*

Therefore, revised search protocol:

(("refugee doctor*") OR ("refugee nurse*") OR ("refugee healthcare professional*")) AND (("NHS") OR ("United Kingdom") OR ("National Health Service") OR ("UK"))

**WEB OF SCIENCE**

(ALL=("refugee doctor*") OR ALL=("refugee nurse*") OR ALL=("refugee pharmacist*") OR ALL=("refugee dentist*") OR ALL=("refugee physiotherapist*") OR ALL=("refugee biomedical scientist*") OR ALL=("refugee healthcare professional*")) AND (ALL=("NHS") OR ALL=("United Kingdom") OR ALL=("National Health Service") OR ALL=("UK"))

# Appendix B: Full Results of Literature Search

**MEDLINER**

1. Adams K, Borman E. Helping refugee doctors. The new asylum act may make it easier to help small numbers of refugee doctors. *BMJ* 2000;320(7239):887-8.
2. Anderson K, Sykes M, Fisher P. Medical students and refugee doctors: learning together. *Medical Education* 2007;41(11):1105-6.
3. Berlin A, Gill P, Eversley J. Refugee doctors in Britain: a wasted resource. *BMJ* 1997;315(7103):264-5.
4. Brooks J. My Questionable Status as a Friendly Enemy Alien: British Responses to Jewish Refugee Nurses 1933 to 1948. *Nurs Hist Rev* 2020;29(1):202-22. doi: https://dx.doi.org/10.1891/1062-8061.29.202
5. Cheeroth S, Ambrose L. A training programme for refugee doctors. *Hosp Med* 2002;63(10):582-3.
6. Cheeroth S, Underwood M, Carter Y, et al. Breaking down barriers for refugee doctors. Secure statutory finding is needed. *BMJ* 2000;321(7261):633-4.
7. Cohn S, Alenya J, Murray K, et al. Experiences and expectations of refugee doctors: Qualitative study. *British Journal of Psychiatry* 2006;189:74-8.
8. Ezsias A. Refugee doctors face enormous difficulty. *BMJ* 1998;316(7137):1095.
9. Hakesley-Brown R. The global nursing workforce: liberating the skills of refugee nurses. *International Nursing Review* 2005;52(4):241-2.
10. Hussain T, Arnold S. Integrating refugee doctors into the NHS. *Annals of the Royal College of Surgeons of England* 2004;86(4):325-8.
11. Leaver L. Overseas-trained refugee doctors. *Medical Education* 2003;37(1):77.
12. Leblanc Y, Bourgeault IL, Neiterman E. Comparing approaches to integrating refugee and asylum-seeking healthcare professionals in Canada and the UK. *Healthcare Policy = Politiques de sante* 2013;9(Spec Issue):126-38.
13. Mayor S. UK helps refugee doctors to practise in NHS. *BMJ* 2000;321(7270):1178.
14. Millar B. Refugee doctors. Long and winding road. *Health Serv J* 2001;111(5741):26-9.
15. Parker R. Breaking down barriers for refugee doctors. Doctors can qualify in the United Kingdom. *BMJ* 2000;321(7261):633.
16. Rosenthal J, Singh S. Overseas Doctor Training Scheme. Plight of refugee doctors. *BMJ* 1994;309(6950):339.
17. Rumani G. Refugee doctors find it hard to get back into practice. *BMJ* 2001;322(7290):862.
18. Stewart E, Nicholas S. Refugee doctors in the United Kingdom. *BMJ* 2002;325(7373):S166.
19. Stewart J. Angels or aliens? Refugee nurses in Britain, 1938 to 1942. *Medical History* 2003;47(2):149-72.
20. Trewby PN. '... a stranger in a strange land': the plight of refugee doctors in the UK. *Clinical Medicine* 2005;5(4):317-9.

**EMBASE**

1. Berlin A, Gill P, Eversley J. Refugee doctors in Britain: A wasted resource. Helping them would help the health service. *British Medical Journal* 1997;315(7103):264-65.
2. Brooks J. My Questionable Status as a Friendly Enemy Alien: British Responses to Jewish Refugee Nurses 1933 to 1948. *Nursing history review : official journal of the American Association for the History of Nursing* 2020;29(1):202-22. doi: https://dx.doi.org/10.1891/1062-8061.29.202
3. Cheeroth S, Ambrose L. A training programme for refugee doctors. *Hospital medicine (London, England : 1998)* 2002;63(10):582-83. doi: http://dx.doi.org/10.12968/hosp.2002.63.10.1926
4. Cheeroth S, Underwood M, Carter Y, et al. Breaking down barriers for refugee doctors. Secure statutory finding is needed. *BMJ (Clinical research ed)* 2000;321(7261):633-34.
5. Cohn S, Alenya J, Murray K, et al. Experiences and expectations of refugee doctors. *British Journal of Psychiatry* 2006;189(JULY):74-78. doi: http://dx.doi.org/10.1192/bjp.bp.105.010975
6. Eastwood JB, Emmett L, Cappuccio FP, et al. Re-training refugee and other overseas doctors: Re-qualification through the United Examining Board examination. *Clinical Medicine, Journal of the Royal College of Physicians of London* 2006;6(1):51-56. doi: http://dx.doi.org/10.7861/clinmedicine.6-1-51
7. Hakesley-Brown R. The global nursing workforce: Liberating the skills of refugee nurses. *International Nursing Review* 2005;52(4):241-42. doi: http://dx.doi.org/10.1111/j.1466-7657.2005.00468.x
8. Hilton C. Refugee doctors and the development of psychiatry [3]. *British Journal of Psychiatry* 2006;189(OCT.):383-84. doi: http://dx.doi.org/10.1192/bjp.189.4.383b
9. Hussain T, Arnold S. Integrating refugee doctors into the NHS [1]. *Annals of the Royal College of Surgeons of England* 2004;86(4):325. doi: http://dx.doi.org/10.1308/147870804443
10. Jackson R. Karl Koenig: The Austrian refugee doctor who founded a worldwide movement in Scotland. *Scottish Medical Journal* 2013;58(2):124-27. doi: http://dx.doi.org/10.1177/0036933013482675
11. Jalil M, Freeman R, Brigley S. How to... Support refugee doctors. *Education for Primary Care* 2007;18(6):759-62. doi: http://dx.doi.org/10.1080/14739879.2007.11493615
12. Leaver LB. Overseas-trained refugee doctors [2]. *Medical Education* 2003;37(1):77. doi: http://dx.doi.org/10.1046/j.1365-2923.2003.00022.x
13. LeBlanc Y, Bourgeault IL, Neiterman E. Comparing approaches to integrating refugee and asylum-seeking healthcare professionals in Canada and the UK. [French]. *Healthcare Policy* 2013;9(SPEC. ISSUE):126-38. doi: http://dx.doi.org/10.12927/hcpol.2013.23596
14. Mayor S. UK helps refugee doctors to practise in NHS. *BMJ (Clinical research ed)* 2000;321(7270):1178.
15. Millar B. Refugee doctors. Long and winding road. *The Health service journal* 2001;111(5741):26-29.
16. Ong YL, Gayen A. Helping refugee doctors get their first jobs: the pan-London clinical attachment scheme. *Hospital medicine (London, England : 1998)* 2003;64(8):488-90.
17. Ong YL, Trafford P, Paice E, et al. Investing in learning and training refugee doctors. *Clinical Teacher* 2010;7(2):131-35. doi: http://dx.doi.org/10.1111/j.1743-498X.2010.00366.x
18. Parker R. Breaking down barriers for refugee doctors. Doctors can qualify in the United Kingdom. *BMJ (Clinical research ed)* 2000;321(7261):633. doi: http://dx.doi.org/10.1136/bmj.321.7261.633
19. Parker R, Cheeroth S, Underwood M, et al. Breaking down barriers for refugee doctors [2] (multiple letters). *British Medical Journal* 2000;321(7261):633-34.
20. Rosenthal J, Singh S. Overseas Doctor Training Scheme. Plight of refugee doctors. *BMJ (Clinical research ed)* 1994;309(6950):339.
21. Rosenthal J, Singh S. Overseas Doctors Training Scheme. Plight of refugee doctors [10]. *British Medical Journal* 1994;309(6950):339.
22. Rumani G. Refugee doctors find it hard to get back into practice [7]. *British Medical Journal* 2001;322(7290):862.
23. Sapper H. Refugee doctors: Filling in some background. *North and West London Journal of General Practice* 2003;9(1):6.
24. Sinclair JMA, Latifi AH, Latifi AW. Refugee doctors as doctors' assistants in psychiatry. *Psychiatric Bulletin* 2006;30(11):430-31. doi: http://dx.doi.org/10.1192/pb.30.11.430
25. Stewart E, Nicholas S. Refugee doctors in the United Kingdom. *BMJ (Clinical research ed)* 2002;325(7373):S166.
26. Stewart J. Angels or aliens? Refugee nurses in Britain, 1938 to 1942. *Medical history* 2003;47(2):149-72.
27. Trewby PN. '... a stranger in a strange land': The plight of refugee doctors in the UK. *Clinical Medicine, Journal of the Royal College of Physicians of London* 2005;5(4):317-19. doi: http://dx.doi.org/10.7861/clinmedicine.5-4-317

**PubMed**

1. Adams K, Borman E. Helping refugee doctors. The new asylum act may make it easier to help small numbers of refugee doctors. *Bmj* 2000;320(7239):887-8. doi: 10.1136/bmj.320.7239.887 [published Online First: 2000/03/31]
2. Anderson K, Sykes M, Fisher P. Medical students and refugee doctors: learning together. *Med Educ* 2007;41(11):1105-6. doi: 10.1111/j.1365-2923.2007.02886.x [published Online First: 2007/09/22]
3. Berlin A, Gill P, Eversley J. Refugee doctors in Britain: a wasted resource. *Bmj* 1997;315(7103):264-5. doi: 10.1136/bmj.315.7103.264 [published Online First: 1997/08/02]
4. Bird J. Welcoming refugee nurses. *Br J Nurs* 2021;30(17):S3. doi: 10.12968/bjon.2021.30.17.S3 [published Online First: 2021/10/05]
5. Brooks J. My Questionable Status as a Friendly Enemy Alien: British Responses to Jewish Refugee Nurses 1933 to 1948. *Nurs Hist Rev* 2020;29(1):202-22. doi: 10.1891/1062-8061.29.202 [published Online First: 2020/12/29]
6. Butt MF, Salmon L, Mulamehic F, et al. Integrating Refugee Healthcare Professionals In The UK National Health Service: Experience From A Multi-Agency Collaboration. *Adv Med Educ Pract* 2019;10:891-96. doi: 10.2147/amep.S213543 [published Online First: 2019/11/22]
7. Cheeroth S, Ambrose L. A training programme for refugee doctors. *Hosp Med* 2002;63(10):582-3. doi: 10.12968/hosp.2002.63.10.1926 [published Online First: 2002/11/09]
8. Cheeroth S, Goraya A. Refugee doctors. *Bmj* 2000;321(7267):S2-s7267. doi: 10.1136/bmj.321.7267.s2-7267 [published Online First: 2000/10/20]
9. Cheeroth S, Underwood M, Carter Y, et al. Breaking down barriers for refugee doctors. Secure statutory finding is needed. *Bmj* 2000;321(7261):633-4. [published Online First: 2000/10/07]
10. Chen MI, von Roenne A, Souare Y, et al. Reproductive health for refugees by refugees in Guinea II: sexually transmitted infections. *Confl Health* 2008;2:14. doi: 10.1186/1752-1505-2-14 [published Online First: 2008/10/25]
11. Cohn S, Alenya J, Murray K, et al. Experiences and expectations of refugee doctors: Qualitative study. *Br J Psychiatry* 2006;189:74-8. doi: 10.1192/bjp.bp.105.010975 [published Online First: 2006/07/04]
12. Eastwood JB, Emmett L, Cappuccio FP, et al. Re-training refugee and other overseas doctors: re-qualification through the United Examining Board examination. *Clin Med (Lond)* 2006;6(1):51-6. doi: 10.7861/clinmedicine.6-1-51 [published Online First: 2006/03/09]
13. Ezsias A. Refugee doctors face enormous difficulty. *Bmj* 1998;316(7137):1095. doi: 10.1136/bmj.316.7137.1095 [published Online First: 1998/04/29]
14. Hakesley-Brown R. The global nursing workforce: liberating the skills of refugee nurses. *Int Nurs Rev* 2005;52(4):241-2. doi: 10.1111/j.1466-7657.2005.00468.x [published Online First: 2005/10/22]
15. Hakesley-Brown R. Silver service. *Nurs Stand* 2006;20(36):70-1. doi: 10.7748/ns2006.05.20.36.70.c4150 [published Online First: 2006/06/08]
16. Hussain T, Arnold S. Integrating refugee doctors into the NHS. *Ann R Coll Surg Engl* 2004;86(4):325-8. doi: 10.1308/147870804443 [published Online First: 2004/07/09]
17. Jackson R. Karl Koenig: the Austrian refugee doctor who founded a worldwide movement in Scotland. *Scott Med J* 2013;58(2):124-7. doi: 10.1177/0036933013482675 [published Online First: 2013/06/04]
18. Kmietowicz Z. Swapping scrubbing brushes for stethoscopes. *Bmj* 2007;334(7597):768. doi: 10.1136/bmj.39175.595486.DB [published Online First: 2007/04/14]
19. Leaver L. Overseas-trained refugee doctors. *Med Educ* 2003;37(1):77. doi: 10.1046/j.1365-2923.2003.00022.x [published Online First: 2003/01/22]
20. Leblanc Y, Bourgeault IL, Neiterman E. Comparing approaches to integrating refugee and asylum-seeking healthcare professionals in Canada and the UK. *Healthc Policy* 2013;9(Spec Issue):126-38. [published Online First: 2014/01/17]
21. Mayor S. UK helps refugee doctors to practise in NHS. *Bmj* 2000;321(7270):1178. [published Online First: 2000/11/10]
22. Millar B. Refugee doctors. Long and winding road. *Health Serv J* 2001;111(5741):26-9. [published Online First: 2001/03/10]
23. Nickless SJ. Refugee doctors-from medical support worker to a fulfilling NHS career. *Bmj* 2022;376:o54. doi: 10.1136/bmj.o54 [published Online First: 2022/01/14]
24. Ong YL, Gayen A. Helping refugee doctors get their first jobs: the pan-London clinical attachment scheme. *Hosp Med* 2003;64(8):488-90. doi: 10.12968/hosp.2003.64.8.2265 [published Online First: 2003/09/10]
25. Ong YL, Trafford P, Paice E, et al. Investing in learning and training refugee doctors. *Clin Teach* 2010;7(2):131-5. doi: 10.1111/j.1743-498X.2010.00366.x [published Online First: 2010/12/08]
26. Parker R. Breaking down barriers for refugee doctors. Doctors can qualify in the United Kingdom. *Bmj* 2000;321(7261):633. [published Online First: 2000/10/07]
27. Rosenthal J, Singh S. Overseas Doctor Training Scheme. Plight of refugee doctors. *Bmj* 1994;309(6950):339. [published Online First: 1994/07/30]
28. Rumani G. Refugee doctors find it hard to get back into practice. *Bmj* 2001;322(7290):862. [published Online First: 2001/04/06]
29. Shah R, Moodambail A, Alam M, et al. An evaluation of the CAPS refugee doctor scheme in London - a survey of outcomes. *Educ Prim Care* 2021;32(2):100-03. doi: 10.1080/14739879.2020.1857662 [published Online First: 2020/12/30]
30. Stewart E, Nicholas S. Refugee doctors in the United Kingdom. *Bmj* 2002;325(7373):S166. doi: 10.1136/bmj.325.7373.s166 [published Online First: 2002/11/16]
31. Stewart J. Angels or aliens? Refugee nurses in Britain, 1938 to 1942. *Med Hist* 2003;47(2):149-72. [published Online First: 2003/05/21]
32. Trewby PN. '... a stranger in a strange land': the plight of refugee doctors in the UK. *Clin Med (Lond)* 2005;5(4):317-9. doi: 10.7861/clinmedicine.5-4-317 [published Online First: 2005/09/06]

**WEB OF KNOWLEDGE**

1. Refugee doctors in United Kingdom need help. *BMJ-British Medical Journal* 1999;318(7186):815-15.
2. Adams K, Borman E. Helping refugee doctors - The new asylum act may make it easier to help small numbers of refugee doctors. *British Medical Journal* 2000;320(7239):887-88. doi: 10.1136/bmj.320.7239.887
3. Anderson K, Sykes M, Fisher P. Medical students and refugee doctors: learning together. *Medical Education* 2007;41(11):1105-06. doi: 10.1111/j.1365-2923.2007.02886.x
4. Butler C, Al Sharou K. Beyond medical practice: cultural and linguistic training of refugee doctors for integration and employment in the United Kingdom. *Eur J Public Health* 2018;28:71-71.
5. Butler C, Al Sharou K. Voices of Refugee Doctors in the United Kingdom: An Exploration of Their Linguistic and Cultural Needs and Aspirations. London: Bloomsbury Publ Inc 2020.
6. Butt MF, Salmon L, Mulamehic F, et al. Integrating Refugee Healthcare Professionals In The UK National Health Service: Experience From A Multi-Agency Collaboration. *Adv Med Educ Pract* 2019;10:891-96. doi: 10.2147/amep.S213543
7. Cohn S, Alenya J, Murray K, et al. Experiences and expectations of refugee doctors - Qualitative study. *British Journal of Psychiatry* 2006;189:74-78. doi: 10.1192/bjp.bp.105.010975
8. Cross D. REFUGEE HEALTHCARE PROFESSIONALS EDUCATION AND TRAINING - REACHE NORTH WEST AS A CASE STUDY. In: Sengupta E, Blessinger P, eds. Refugee Education: Integration and Acceptance of Refugees in Mainstream Society. Bingley: Emerald Group Publishing Ltd 2018:47-64.
9. Eastwood JB, Emmett L, Cappuccio FP, et al. Re-training refugee and other overseas doctors: re-qualification through the United Examining Board examination. *Clinical Medicine* 2006;6(1):51-56. doi: 10.7861/clinmedicine.6-1-51
10. Fisher P. Film on NHS for new immigrants. *Br J Gen Pract* 2009;59(561):289-90. doi: 10.3399/bjgp09X420374
11. Gavin M, Esmail A. Solving the recruitment crisis in UK general practice: Time to consider physician assistants? *Soc Policy Adm* 2002;36(1):76-89. doi: 10.1111/1467-9515.00271
12. Hilton C. Refugee doctors and the development of psychiatry. *British Journal of Psychiatry* 2006;189:383-84. doi: 10.1192/bjp.189.4.383b
13. Hussain T, Arnold S. Integrating refugee doctors into the NHS. *Annals of the Royal College of Surgeons of England* 2004;86(4):325-25.
14. Mahase E. Covid-19: Refugee doctors join NHS through innovative scheme. *BMJ-British Medical Journal* 2020;375:2. doi: 10.1136/bmj.n2993
15. Mayor S. UK helps refugee doctors to practise in NHS. *British Medical Journal* 2000;321(7270):1178-78. doi: 10.1136/bmj.321.7270.1178
16. Nickless SJ. Refugee doctors-from medical support worker to a fulfilling NHS career. *BMJ-British Medical Journal* 2022;376:1. doi: 10.1136/bmj.o54
17. Ong YL, Gayen A. Helping refugee doctors get their first jobs: the pan-London clinical attachment scheme. *Hospital Medicine* 2003;64(8):488-90. doi: 10.12968/hosp.2003.64.8.2265
18. Parker R. Breaking down barriers for refugee doctors - Doctors can qualify in the United Kingdom. *British Medical Journal* 2000;321(7261):633-33. doi: 10.1136/bmj.321.7261.633
19. Pietka-Nykaza E. 'I Want to Do Anything which Is Decent and Relates to My Profession': Refugee Doctors' and Teachers' Strategies of Re-Entering Their Professions in the UK. *Journal of Refugee Studies* 2015;28(4):523-43. doi: 10.1093/jrs/fev008
20. Qureshi B. Refugee doctors. Support, development and integration in the NHS. *J R Soc Promot Health* 2004;124(6):287-87. doi: 10.1177/146642400412400620
21. Roberts GJ. Tall trees; weak roots? A model of barriers to English language proficiency confronting displaced medical healthcare professionals. *Language Teaching Research*:17. doi: 10.1177/1362168820968366
22. Shah RP, Moodambail A, Alam M, et al. An evaluation of the CAPS refugee doctor scheme in London - a survey of outcomes. *Education for Primary Care* 2021;32(2):100-03. doi: 10.1080/14739879.2020.1857662
23. Trewby PN. '... a stranger in a strange land': The plight of refugee doctors in the UK. *Clinical Medicine* 2005;5(4):317-19. doi: 10.7861/clinmedicine.5-4-317

**NON-DUPLICATE CITATIONS**

1. **Refugee doctors in United Kingdom need help. *BMJ-British Medical Journal* 1999;318(7186):815-15.**
2. **Adams K, Borman E. Helping refugee doctors. The new asylum act may make it easier to help small numbers of refugee doctors. *BMJ* 2000;320(7239):887-8.**
3. Anderson K, Sykes M, Fisher P. Medical students and refugee doctors: learning together. *Medical Education* 2007;41(11):1105-6.
4. **Berlin A, Gill P, Eversley J. Refugee doctors in Britain: A wasted resource. Helping them would help the health service. *British Medical Journal* 1997;315(7103):264-65.**
5. **Bird J. Welcoming refugee nurses. *Br J Nurs* 2021;30(17):S3. doi: 10.12968/bjon.2021.30.17.S3 [published Online First: 2021/10/05]**
6. **Brooks J. My Questionable Status as a Friendly Enemy Alien: British Responses to Jewish Refugee Nurses 1933 to 1948. *Nursing history review : official journal of the American Association for the History of Nursing* 2020;29(1):202-22. doi: https://dx.doi.org/10.1891/1062-8061.29.202**
7. Butler C, Al Sharou K. Beyond medical practice: cultural and linguistic training of refugee doctors for integration and employment in the United Kingdom. *Eur J Public Health* 2018;28:71-71.
8. **Butler C, Al Sharou K. Voices of Refugee Doctors in the United Kingdom: An Exploration of Their Linguistic and Cultural Needs and Aspirations. London: Bloomsbury Publ Inc 2020.**
9. Butt MF, Salmon L, Mulamehic F, et al. Integrating Refugee Healthcare Professionals In The UK National Health Service: Experience From A Multi-Agency Collaboration. *Adv Med Educ Pract* 2019;10:891-96. doi: 10.2147/amep.S213543 [published Online First: 2019/11/22]
10. Cheeroth S, Ambrose L. A training programme for refugee doctors. *Hospital medicine (London, England : 1998)* 2002;63(10):582-83. doi: http://dx.doi.org/10.12968/hosp.2002.63.10.1926
11. **Cheeroth S, Goraya A. Refugee doctors. *Bmj* 2000;321(7267):S2-s7267. doi: 10.1136/bmj.321.7267.s2-7267 [published Online First: 2000/10/20]**
12. **Cheeroth S, Underwood M, Carter Y, et al. Breaking down barriers for refugee doctors. Secure statutory finding is needed. *BMJ (Clinical research ed)* 2000;321(7261):633-34.**
13. **Chen MI, von Roenne A, Souare Y, et al. Reproductive health for refugees by refugees in Guinea II: sexually transmitted infections. *Confl Health* 2008;2:14. doi: 10.1186/1752-1505-2-14 [published Online First: 2008/10/25]**
14. Cohn S, Alenya J, Murray K, et al. Experiences and expectations of refugee doctors: Qualitative study. *British Journal of Psychiatry* 2006;189:74-8.
15. **Cross D. REFUGEE HEALTHCARE PROFESSIONALS EDUCATION AND TRAINING - REACHE NORTH WEST AS A CASE STUDY. In: Sengupta E, Blessinger P, eds. Refugee Education: Integration and Acceptance of Refugees in Mainstream Society. Bingley: Emerald Group Publishing Ltd 2018:47-64.**
16. Eastwood JB, Emmett L, Cappuccio FP, et al. Re-training refugee and other overseas doctors: Re-qualification through the United Examining Board examination. *Clinical Medicine, Journal of the Royal College of Physicians of London* 2006;6(1):51-56. doi: http://dx.doi.org/10.7861/clinmedicine.6-1-51
17. **Ezsias A. Refugee doctors face enormous difficulty. *BMJ* 1998;316(7137):1095.**
18. **Fisher P. Film on NHS for new immigrants. *Br J Gen Pract* 2009;59(561):289-90. doi: 10.3399/bjgp09X420374**
19. Gavin M, Esmail A. Solving the recruitment crisis in UK general practice: Time to consider physician assistants? *Soc Policy Adm* 2002;36(1):76-89. doi: 10.1111/1467-9515.00271
20. **Hakesley-Brown R. The global nursing workforce: Liberating the skills of refugee nurses. *International Nursing Review* 2005;52(4):241-42. doi: http://dx.doi.org/10.1111/j.1466-7657.2005.00468.x**
21. **Hakesley-Brown R. Silver service. *Nurs Stand* 2006;20(36):70-1. doi: 10.7748/ns2006.05.20.36.70.c4150 [published Online First: 2006/06/08]**
22. **Hilton C. Refugee doctors and the development of psychiatry. *British Journal of Psychiatry* 2006;189:383-84. doi: 10.1192/bjp.189.4.383b**
23. **Hussain T, Arnold S. Integrating refugee doctors into the NHS. *Annals of the Royal College of Surgeons of England* 2004;86(4):325-8.**
24. **Jackson R. Karl Koenig: The Austrian refugee doctor who founded a worldwide movement in Scotland. *Scottish Medical Journal* 2013;58(2):124-27. doi: http://dx.doi.org/10.1177/0036933013482675**
25. Jalil M, Freeman R, Brigley S. How to... Support refugee doctors. *Education for Primary Care* 2007;18(6):759-62. doi: http://dx.doi.org/10.1080/14739879.2007.11493615
26. **Kmietowicz Z. Swapping scrubbing brushes for stethoscopes. *Bmj* 2007;334(7597):768. doi: 10.1136/bmj.39175.595486.DB [published Online First: 2007/04/14]**
27. **Leaver L. Overseas-trained refugee doctors. *Medical Education* 2003;37(1):77.**
28. Leblanc Y, Bourgeault IL, Neiterman E. Comparing approaches to integrating refugee and asylum-seeking healthcare professionals in Canada and the UK. *Healthcare Policy = Politiques de sante* 2013;9(Spec Issue):126-38.
29. **Mahase E. Covid-19: Refugee doctors join NHS through innovative scheme. *BMJ-British Medical Journal* 2020;375:2. doi: 10.1136/bmj.n2993**
30. **Mayor S. UK helps refugee doctors to practise in NHS. *BMJ (Clinical research ed)* 2000;321(7270):1178.**
31. **Millar B. Refugee doctors. Long and winding road. *The Health service journal* 2001;111(5741):26-29.**
32. **Nickless SJ. Refugee doctors-from medical support worker to a fulfilling NHS career. *Bmj* 2022;376:o54. doi: 10.1136/bmj.o54 [published Online First: 2022/01/14]**
33. Ong YL, Gayen A. Helping refugee doctors get their first jobs: the pan-London clinical attachment scheme. *Hospital medicine (London, England : 1998)* 2003;64(8):488-90.
34. Ong YL, Trafford P, Paice E, et al. Investing in learning and training refugee doctors. *Clinical Teacher* 2010;7(2):131-35. doi: http://dx.doi.org/10.1111/j.1743-498X.2010.00366.x
35. **Parker R. Breaking down barriers for refugee doctors. Doctors can qualify in the United Kingdom. *BMJ (Clinical research ed)* 2000;321(7261):633. doi: http://dx.doi.org/10.1136/bmj.321.7261.633**
36. Pietka-Nykaza E. 'I Want to Do Anything which Is Decent and Relates to My Profession': Refugee Doctors' and Teachers' Strategies of Re-Entering Their Professions in the UK. *Journal of Refugee Studies* 2015;28(4):523-43. doi: 10.1093/jrs/fev008
37. **Qureshi B. Refugee doctors. Support, development and integration in the NHS. *J R Soc Promot Health* 2004;124(6):287-87. doi: 10.1177/146642400412400620**
38. Roberts GJ. Tall trees; weak roots? A model of barriers to English language proficiency confronting displaced medical healthcare professionals. *Language Teaching Research*:17. doi: 10.1177/1362168820968366
39. **Rosenthal J, Singh S. Overseas Doctor Training Scheme. Plight of refugee doctors. *BMJ (Clinical research ed)* 1994;309(6950):339.**
40. **Rumani G. Refugee doctors find it hard to get back into practice. *BMJ* 2001;322(7290):862.**
41. **Sapper H. Refugee doctors: Filling in some background. *North and West London Journal of General Practice* 2003;9(1):6.**
42. Shah RP, Moodambail A, Alam M, et al. An evaluation of the CAPS refugee doctor scheme in London - a survey of outcomes. *Education for Primary Care* 2021;32(2):100-03. doi: 10.1080/14739879.2020.1857662
43. Sinclair JMA, Latifi AH, Latifi AW. Refugee doctors as doctors' assistants in psychiatry. *Psychiatric Bulletin* 2006;30(11):430-31. doi: http://dx.doi.org/10.1192/pb.30.11.430
44. Stewart E, Nicholas S. Refugee doctors in the United Kingdom. *BMJ (Clinical research ed)* 2002;325(7373):S166.
45. **Stewart J. Angels or aliens? Refugee nurses in Britain, 1938 to 1942. *Medical history* 2003;47(2):149-72.**
46. Trewby PN. '... a stranger in a strange land': The plight of refugee doctors in the UK. *Clinical Medicine, Journal of the Royal College of Physicians of London* 2005;5(4):317-19. doi: http://dx.doi.org/10.7861/clinmedicine.5-4-317

**AFTER TITLE/ABSTRACT SCREEN (29 EXCLUDED)**

1. Anderson K, Sykes M, Fisher P. Medical students and refugee doctors: learning together. *Medical Education* 2007;41(11):1105-6.
2. **Butler C, Al Sharou K. Beyond medical practice: cultural and linguistic training of refugee doctors for integration and employment in the United Kingdom. *Eur J Public Health* 2018;28:71-71.**
3. Butt MF, Salmon L, Mulamehic F, et al. Integrating Refugee Healthcare Professionals In The UK National Health Service: Experience From A Multi-Agency Collaboration. *Adv Med Educ Pract* 2019;10:891-96. doi: 10.2147/amep.S213543 [published Online First: 2019/11/22]
4. **Cheeroth S, Ambrose L. A training programme for refugee doctors. *Hospital medicine (London, England : 1998)* 2002;63(10):582-83. doi: http://dx.doi.org/10.12968/hosp.2002.63.10.1926**
5. Cohn S, Alenya J, Murray K, et al. Experiences and expectations of refugee doctors: Qualitative study. *British Journal of Psychiatry* 2006;189:74-8.
6. Eastwood JB, Emmett L, Cappuccio FP, et al. Re-training refugee and other overseas doctors: Re-qualification through the United Examining Board examination. *Clinical Medicine, Journal of the Royal College of Physicians of London* 2006;6(1):51-56. doi: http://dx.doi.org/10.7861/clinmedicine.6-1-51
7. Gavin M, Esmail A. Solving the recruitment crisis in UK general practice: Time to consider physician assistants? *Soc Policy Adm* 2002;36(1):76-89. doi: 10.1111/1467-9515.00271
8. **Jalil M, Freeman R, Brigley S. How to... Support refugee doctors. *Education for Primary Care* 2007;18(6):759-62. doi: http://dx.doi.org/10.1080/14739879.2007.11493615**
9. Leblanc Y, Bourgeault IL, Neiterman E. Comparing approaches to integrating refugee and asylum-seeking healthcare professionals in Canada and the UK. *Healthcare Policy = Politiques de sante* 2013;9(Spec Issue):126-38.
10. Ong YL, Gayen A. Helping refugee doctors get their first jobs: the pan-London clinical attachment scheme. *Hospital medicine (London, England : 1998)* 2003;64(8):488-90.
11. Ong YL, Trafford P, Paice E, et al. Investing in learning and training refugee doctors. *Clinical Teacher* 2010;7(2):131-35. doi: http://dx.doi.org/10.1111/j.1743-498X.2010.00366.x
12. Pietka-Nykaza E. 'I Want to Do Anything which Is Decent and Relates to My Profession': Refugee Doctors' and Teachers' Strategies of Re-Entering Their Professions in the UK. *Journal of Refugee Studies* 2015;28(4):523-43. doi: 10.1093/jrs/fev008
13. Roberts GJ. Tall trees; weak roots? A model of barriers to English language proficiency confronting displaced medical healthcare professionals. *Language Teaching Research*:17. doi: 10.1177/1362168820968366
14. Shah RP, Moodambail A, Alam M, et al. An evaluation of the CAPS refugee doctor scheme in London - a survey of outcomes. *Education for Primary Care* 2021;32(2):100-03. doi: 10.1080/14739879.2020.1857662
15. Sinclair JMA, Latifi AH, Latifi AW. Refugee doctors as doctors' assistants in psychiatry. *Psychiatric Bulletin* 2006;30(11):430-31. doi: http://dx.doi.org/10.1192/pb.30.11.430
16. Stewart E, Nicholas S. Refugee doctors in the United Kingdom. *BMJ (Clinical research ed)* 2002;325(7373):S166.
17. **Trewby PN. '... a stranger in a strange land': The plight of refugee doctors in the UK. *Clinical Medicine, Journal of the Royal College of Physicians of London* 2005;5(4):317-19. doi:** [**http://dx.doi.org/10.7861/clinmedicine.5-4-317**](http://dx.doi.org/10.7861/clinmedicine.5-4-317)

**AFTER FULL TEXT SCREEN (4 EXCLUDED: 3 VIA SCREENING, 1 FOR LACKING FULL ACCESS)**

1. Anderson K, Sykes M, Fisher P. Medical students and refugee doctors: learning together. *Medical Education* 2007;41(11):1105-6.
2. Butt MF, Salmon L, Mulamehic F, et al. Integrating Refugee Healthcare Professionals In The UK National Health Service: Experience From A Multi-Agency Collaboration. *Adv Med Educ Pract* 2019;10:891-96. doi: 10.2147/AMEP.S213543 [published Online First: 2019/11/22]
3. Cohn S, Alenya J, Murray K, et al. Experiences and expectations of refugee doctors: Qualitative study. *Br J Psychiatry* 2006;189:74-8. doi: 10.1192/bjp.bp.105.010975 [published Online First: 2006/07/04]
4. Eastwood JB, Emmett L, Cappuccio F, et al. Re-training refugee and other overseas doctors: re-qualification through the United Examining Board examination. *Clinical Medicine* 2006;6(1):51-56. doi: 10.7861/clinmedicine.6-1-51
5. Gavin M, Esmail A. Solving the Recruitment Crisis in UK General Practice: Time to Consider Physician Assistants? *Social Policy and Administration* 2002;36(1):76-89. doi: 10.1111/1467-9515.00271
6. Leblanc Y, Bourgeault IL, Neiterman E. Comparing approaches to integrating refugee and asylum-seeking healthcare professionals in Canada and the UK. *Healthc Policy* 2013;9(Spec Issue):126-38. [published Online First: 2014/01/17]
7. Ong Y-L, Gayen A. Helping refugee doctors get their first jobs: the pan-London clinical attachment scheme. *Hospital Medicine* 2003;64(8):488-90. doi: 10.12968/hosp.2003.64.8.2265
8. Ong YL, Trafford P, Paice E, et al. Investing in learning and training refugee doctors. *The Clinical Teacher* 2010;7(2):131-35. doi: 10.1111/j.1743-498x.2010.00366.x
9. Piętka-Nykaza E. ‘I Want to Do Anything which Is Decent and Relates to My Profession’: Refugee Doctors’ and Teachers’ Strategies of Re-Entering Their Professions in the UK. *Journal of Refugee Studies* 2015;28(4):523-43. doi: 10.1093/jrs/fev008
10. Roberts GJ. Tall trees; weak roots? A model of barriers to English language proficiency confronting displaced medical healthcare professionals. *Language Teaching Research* 2020:136216882096836. doi: 10.1177/1362168820968366
11. Shah R, Moodambail A, Alam M, et al. An evaluation of the CAPS refugee doctor scheme in London – a survey of outcomes. *Education for Primary Care* 2021;32(2):100-03. doi: 10.1080/14739879.2020.1857662
12. Sinclair JMA, Latifi AH, Latifi AW. Refugee doctors as doctors' assistants in psychiatry. *Psychiatric Bulletin* 2006;30(11):430-31. doi: 10.1192/pb.30.11.430
13. Stewart E, Nicholas S. Refugee doctors in the United Kingdom. *BMJ* 2002;325(7373):S166. doi: 10.1136/bmj.325.7373.s166 [published Online First: 2002/11/16]

# Appendix C: Full Results of Data Analysis

| Study | Barriers | Initiative | Improvements |
| --- | --- | --- | --- |
| Anderson |  | - Informal OSCE preparation practice between medical students and refugee doctors - Helpful for improving the idiomatic and slang language skills of refugee doctors, as well as improving their familiarity of the UK doctor-patient relationship, their confidence in interacting with native British people, and a sense of social support |  |
| Butt | - Interpersonal: Negotiating working relationships with other professionals - Structural: must learn new medicolegal frameworks, training systems, skills guidelines - Personal/Unique to RASHPs: may have faced persecution and trauma in homeland, lost relevant paperwork, now in unfamiliar healthcare system, need to have successfully applied for asylum to receive refugee status => system may enforce long-term unemployment => lack of confidence, deskilling | - Training program to help integrate refugee healthcare professionals into the NHS - Can be helpful for improving employment outcomes for refugee healthcare professionals, but it appears that a large proportion struggle to find employment in their original area of training - Limited by loss to follow-up, unknown employment history, and lack of control group |  |
| Cohn | - Personal barriers: establishing semi-permanent accommodation, managing finances, ongoing familial responsibilities, language, psychological impacts of struggling to succeed in UK system - Structural barriers: immigration issues, accessing information and progressing through the system, feeling of always being placed behind local medical professionals, marginalised in society, experiences of racism and discrimination - Refugee support organisations helpful, but limited in scope and quality and contribute to homogenisation of refugee doctors and the negative impacts of the label |  |  |
| Eastwood | - Unique to RASHPs: migrating in the form of a hasty flight from oppression rather than a planned like most non-refugee internationally, resulting in longer breaks in practice for RASHPs (shown with quantitative data), all making passing the PLAB much more challenging in comparison to non-refugee counterparts - Years since last practicing for refugee doctors: mean of 4.5 years (versus 3.5 for non-refugee counterparts) - As of 2006: only 77 of the 1047 doctors on the BMA/Refugee Council database are known to be employed as doctors in the NHS | - Formal training courses and exam preparation for refugee doctors - May be a cost-effective method of helping refugee doctors successfully enter the workforce - Challenge in collecting data about refugee doctors, as many are reluctant to keep in touch with authorities/identify themselves as refugees | - There has been investment by the Department of Health into helping refugee doctors pass the PLAB, but no data on outcome |
| Gavin |  |  | - Suggests siphoning refugee doctors into PA training and introducing into GP workforce, but no data on such programs |
| Leblanc | - Personal and structural barriers: economically compromised due to legal restrictions on mobility and access to employment, worse for female refugees, worse depending on individual circumstances eg. experiences of torture, loss of family members, trauma, health problems - Unique to RASHPs: Lacking proof of credentials eg. references from medical school, portfolios of evidence to demonstrate specialist training; lacking recognition of credentials for those with proof, limited options for retraining, difficulty finding orientation placements, lack of support; underemployment; discrimination; legal restrictions that may limit access to paid employment; significant gap in training => many need to retrain, but refugees often lack the financial means, programs very time-consuming; many forced to abandon their original career and seek employment in other healthcare roles or other fields entirely (many are told to “manage” their expectations and consider more “realistic” career paths); stigma surrounding being known as a refugee doctor can be a significant drawback; passing proficiency exams does not guarantee an individual has reached level that satisfies employment requirements; individual skill level, availability of training and mentorship, generic design of language assessment tool |  | - Language training must be redesigned and tailored to the needs of RASHPs, but limited investment so far |
| Ong (2003) | - Unique to RASHPs: nearly all participating refugee doctors scored symptoms on the Post Traumatic Stress Disorder (PTSD) scale, with some scoring enough to cross the threshold for a clinical diagnosis of PTSD | - Unpaid clinical attachment scheme for “job ready” refugee doctors - Only a minority of doctors who partook in the scheme went on to find substantive employment |  |
| Ong (2010) | - Interpersonal barriers: Cultural adaptation and relating to patients, changing their communication styles, adjusting to teamwork approach in UK and equality amongst professional groups (some were seen as arrogant and demanding) - Structural barriers: different learning styles making it difficult to adjust to style of education being offered, experiences of discrimination (eg. racism, sexism, ageism) | - Ring-fenced salaried posts with intensive education schedule - Greatly enhances likelihood of gaining substantive employment in comparison to simple clinical attachments, but also require more substantial investment - Challenge in collecting data about refugee doctors, as many are reluctant to keep in touch with authorities/identify themselves as refugees |  |
| Pietka-Nykaza | - Structural barriers: Legal restriction on rights (including to work) for asylum seekers, which last from months to years, leading to disturbance in work history/gap in CV; being granted only a five-year term instead of indefinite leave to remain in UK also hinders long-term employment goals and plans - Qualifications not seen as equivalent to UK standards, IELTS and PLAB exams can be challenging   - individual circumstances: mental health problems related to forced exile and violence, negative representations of asylum seekers and refugees in media and public discourse   - Lacking proof of credentials or lacking understanding/recognition of their credentials   - Qualitative data: interviews of refugee doctors and teachers => understand how refugees make their decisions on their future professional career in the UK and how they justify it, each of the four depends on demographics of refugee and their personal circumstances - Decision making process for RASHPs confronted with these barriers: Acceptance, Compromise, Ambivalence, Withdrawal - Career paths not always the result of their individual choices, responses to individual barriers, opportunities, and personal dilemmas |  |  |
| Roberts | - As of 2008: of registered refugee doctors, 14% currently practicing medicine, 20% job ready but not working, 7% passed PLAB 1, but not yet PLAB 2, 14% passed IELTS, but not PLAB yet, 45% have not passed IELTS |  | - Language tuition must be tailored to the specific needs of the group eg. medical English |
| Shah |  | - Ring-fenced salaried posts for refugee doctors with educational support, linguistic resources, pastoral care - Effective in helping refugee doctors obtain substantive employment: 93% of doctors completing CAPS were retained in the NHS - CAPS program thought to help mitigate against several factors (lack of social and cultural capital, relationships, identity and sense of belonging) that contribute to differential attainment between BAME medical practitioners and their white counterparts via fostering relationships within learning sets, support with language, acculturation and careers guidance - Majority of doctors who passed through CAPS are employed in non-training positions, likely related to difficulty getting into specialty training programmes | - Communications skills support was most frequently cited benefit of Professional Support Unit; this can be a focus for future programs - Given that general practice is popular specialty choice for refugee doctors, continued support for them with future programs like CAPS may be significant in addressing the UK’s GP shortage |
| Sinclair |  | - Refugee doctors offered posts as doctors’ assistants in psychiatry - Shows promise in having a positive impact at a personal and institutional level, as well as providing experience and access to training and resources that help refugee doctors work towards registration with the GMC |  |
| Stewart | - Personal barriers: lack of personal and professional networks, problems adapting to different culture, emotional and psychological effects of being undervalued as a doctor in the UK (worsened by prolonged time taken to re-enter profession that causes significant deskilling) - Structural barriers: IELTS as an assessment tool, not tailored to actual language proficiency and skills required to practice medicine, required for many refugee doctors even if they were trained in English, despite their European counterparts who were not trained in English often being exempt from the exam; feelings of local doctors being preferred over refugee doctors for jobs, problems in obtaining employment in training positions for career progression; Experience of prejudice |  |  |

# Appendix D: Quality Assessment

This review applied Lorenc et al.’s adaptation of Hawker et al.’s tool for the assessment of qualitative evidence (3, 4). The original tool involves nine questions for which each can be answered as ‘good’, ‘fair’, ‘poor’, or ‘very poor’ (3). The adapted tool involves the conversion of each answer to a numerical score which can be summed to a total score range of 9-36 points (4). It also provides overall quality grades based on score ranges: high quality (A), 30–36 points; medium quality (B), 24–29 points; low quality (C), 9–24 points (4).

Each selected paper was individually assessed by the lead author to provide an overall numerical score and quality grade. The overall appropriateness of the existing research for answering the research aims was then also considered.

| **Study** | **Abstract/title** | **Introduction/aims** | **Data colleciton** | **Sampling** | **Analysis** | **Ethics/bias** | **Results** | **Generability** | **Implications** | **Total** | **Grade** |
| --- | --- | --- | --- | --- | --- | --- | --- | --- | --- | --- | --- |
| Anderson | 4 | 2 | 2 | 1 | 1 | 1 | 3 | 2 | 2 | 18 | **C** |
| Butt | 4 | 2 | 4 | 4 | 3 | 4 | 4 | 3 | 3 | 31 | **A** |
| Cohn | 4 | 2 | 4 | 4 | 4 | 4 | 4 | 4 | 3 | 33 | **A** |
| Eastwood | 4 | 2 | 3 | 3 | 2 | 1 | 4 | 4 | 2 | 25 | **B** |
| Gavin | 4 | 3 | 1 | 1 | 1 | 2 | 1 | 4 | 4 | 21 | **C** |
| Leblanc | 4 | 2 | 3 | 2 | 3 | 1 | 4 | 4 | 4 | 27 | **B** |
| Ong (2003) | 3 | 2 | 3 | 4 | 2 | 2 | 4 | 3 | 3 | 26 | **B** |
| Ong (2010) | 4 | 3 | 4 | 4 | 2 | 2 | 4 | 3 | 3 | 29 | **B** |
| Pietka-Nykaza | 4 | 4 | 4 | 4 | 4 | 1 | 4 | 4 | 3 | 32 | **A** |
| Roberts | 3 | 4 | 4 | 4 | 4 | 2 | 4 | 4 | 4 | 33 | **A** |
| Shah | 4 | 2 | 4 | 4 | 4 | 3 | 4 | 4 | 3 | 32 | **A** |
| Sinclair | 1 | 2 | 3 | 2 | 1 | 1 | 4 | 2 | 1 | 17 | **C** |
| Stewart | 2 | 2 | 2 | 2 | 1 | 1 | 4 | 2 | 2 | 18 | **C** |

Many studies shared the same issues with data collection. Control groups were not feasible, and many participants were lost to follow-up, likely given the negative experiences RASHPs may have had with authorities and the stigma associated with identifying themselves as “refugee doctors” (12, 13, 15).

The quality of the papers as assessed with the chosen tool showed mixed results. The scores ranged from 17/36 (Sinclair) to 33/36 (Cohn, Roberts). Overall, four papers were assessed as Grade C, four as Grade B, and five as Grade A.

Most papers were strong in their description of results, discussion, and implications of research, but the description of research aims, study methodology, and data analysis less so. Another weak area for many studies was the acknowledgement of ethical considerations and possible effects of bias.
